# Association Between Neutrophil Percentage–Albumin Ratio and Biological Aging in Rheumatoid Arthritis in the United States: A Cross‐Sectional Study of NHANES

**DOI:** 10.1155/mi/9987170

**Published:** 2026-01-16

**Authors:** Yangyu Xu, Hong Zhao, Yuxiang Gao, Li Zhao, Jiannan Han, Rong Li, Zewen Wu, Junkang Zhao, Liyun Zhang

**Affiliations:** ^1^ Rheumatology and Immunology Department, Third Hospital of Shanxi Medical University, Shanxi Bethune Hospital, Shanxi Academy of Medical Sciences, Tongji Shanxi Hospital, Taiyuan, 030032, Shanxi, China; ^2^ School of Public Health, Shanxi Medical University, Taiyuan, 030001, China, sxmu.edu.cn; ^3^ Shanxi Province Clinical Research Center for Dermatologic and Immunologic Diseases (Rheumatic diseases), Taiyuan, China

**Keywords:** inflammatory aging, KDM age, neutrophil percentage-to-albumin ratio (NPAR), NHANES study, phenotypic age, rheumatoid arthritis

## Abstract

**Background:**

The accelerating process of global aging has made the burden of age‐related diseases increasingly severe, and traditional chronological age fails to reflect individual heterogeneity in aging. The neutrophil percentage‐to‐albumin ratio (NPAR), is a multidimensional health assessment index composed of inflammatory markers (neutrophils) and nutritional markers (albumin) to reflect inflammation and nutritional status, has shown unique potential in rheumatoid arthritis (RA) research. However, its association with biological age (BA; such as Klemera–Doubal method [KDM] age and phenotypic age, PhenoAge) has not yet been systematically validated in RA patients. By evaluating NPAR indicators in patients with RA, this study intends to reveal its value as a potential biomarker for predicting biological aging and its acceleration.

**Methods:**

This study was based on the National Health and Nutrition Survey 1999–2018 cycle database, and a cross‐sectional analysis of 1053 adult patients with RA was included. Core variable definitions include: neutrophil–albumin ratio (NPAR) = percentage of neutrophils (%)/albumin (g/dL); BA was calculated by the KDM (including 10 biomarkers) and the PhenoAge algorithm, respectively. Accelerated aging is quantified as the difference between BA and chronological age. The statistical analysis used a multi‐model validation strategy: 1) multivariate linear regression to evaluate the association between NPAR and continuous aging acceleration indicators; 2) the restricted cubic spline (RCS) model explores the nonlinear relationship; 3) stratified subgroup analysis to test for effect heterogeneity. All models were stratified for sociodemographic characteristics (age, sex, and ethnicity), lifestyle factors (smoking, alcohol consumption, and physical activity), and clinical covariates (body mass index [BMI], hypertension, and history of diabetes).

**Results:**

In an analysis of 1053 RA patients in the United States, women accounted for 56.48% and men for 43.52%; the group with the highest NPAR (T3) showed more significant aging characteristics (≥65 years old 34.75%, females 62.80%, and diabetes 27.69%) and higher biological aging acceleration rates (KDM acceleration 42.81% vs. low group 25.81%; PhenoAge acceleration 62.13% vs. 37.74%; all *p*  < 0.001). After adjustment for multiple factors, the BA of KDM increased by 0.86 years for every 1 unit increase in NPAR (95% confidence interval [CI]: 0.39–1.32, *p*  < 0.001), and PhenoAge increased by 1.32 years (95% CI: 0.93–1.71, *p*  < 0.001). Taking the lowest NPAR group (T1) as the reference group, the highest NPAR group (T3) had an increased risk of accelerated aging of KDM by 149% (OR = 2.49, 95% CI: 1.44–4.31), and the risk of PhenoAge increased sharply by 259% (OR = 3.59, 95% CI: 2.17–5.95). Nonrestrictive spline curve analysis further revealed that there was a nonlinear positive correlation between the NPAR index and biological aging. When NPAR > 13.128, the growth rate of biological aging was significantly accelerated (*p* for nonlinear = 0.05), while when NPAR > 14.512, the risk of phenotypic aging accelerated sharply (*p* for nonlinear = 0.002). Area under the curve (AUC) of NPAR in the combined model for predicting biological senescence acceleration was 0.71–0.75.

**Discussion:**

Elevated NPAR is significantly associated with accelerated biological aging in RA patients, and the mechanism may involve neutrophil migration dysfunction, oxidative damage caused by albumin deficiency, and chronic inflammatory pathways (such as NF‐*κ*B activation). As a low‐cost inflammatory marker, NPAR holds promise for integration into clinical aging risk assessment systems to identify high‐risk RA populations requiring early intervention. The study’s limitations include the inability to infer causality due to the cross‐sectional design and a relatively small sample size, necessitating further validation through cohort studies in the future.

## 1. Introduction

As the global population ages, age‐related diseases [1] have become an important public health challenge. Data shows that in 2017, aging‐related diseases accounted for 51.3% of the global adult health burden. While chronological age (actual age) can conveniently reflect aging, the rate of aging may differ among individuals of the same chronological age, and the heterogeneity of physiological functions between individuals tends to increase with age. Using chronological age to reflect the level of aging has certain limitations, so we need better markers to measure individual aging processes, that is, biological age (BA) [2]. Different BA estimation methods, including epigenetic clock [3], phenotypic age (PhenoAge) [4], and Klemera–Doubal method (KDM) [5], may capture different dimensions of aging[6], epigenetic age clocks, such as the Horvath clock, have been used to reveal the phenomenon of accelerated biological aging in rheumatoid arthritis (RA) patients, showing a close association between RA and biological aging[7, 8], the use of “DNA methylation age clocks” based on epigenetic data, such as DNAm GrimAge, allows for molecular‐level prediction of lifespan and disease progression in RA patients [9], the KDM and phenotypic age (PhenoAge) as BAs have attracted much attention for their ability to comprehensively assess multidimensional physiological functions [10].

RA is a systemic autoimmune disease that primarily affects the joints [11] and is the most common form of chronic inflammatory arthritis, characterized primarily by joint destruction [12], affecting approximately 1% of the world’s population [13]. The abnormal activation of synovial fibroblasts leads to the continuous secretion of pro‐inflammatory factors such as IL‐6 and TNF‐*α*, forming an “inflammatory self‐sustaining circuit,” long‐term autoimmune inflammation can induce chronic inflammatory responses and damage tissues, such as synovial hyperplasia and bone destruction, which are causally related to accelerated systemic biological aging [9]. BA acceleration is an effective indicator for predicting a patient’s remaining lifespan. Patients with BA acceleration have a significantly increased risk of complications and a more pronounced reduction in lifespan [7, 14]. BA acceleration may increase the risk of RA, especially in populations with high genetic risk, and may also reduce the expected lifespan of individuals with RA [7] and existing studies have shown that neutrophil/lymphocyte ratio (NLR) and platelet/lymphocyte ratio (PLR) show high specificity (area under the curve [AUC] = 0.78) in differentiating RA from other arthritis [15]. And neutrophil percentage‐to‐albumin ratio (NPAR), as a comprehensive indicator of integrating inflammation and nutritional status, has shown unique potential in RA research in recent years [16, 17], and chronic inflammation, as one of the three core features of aging [18], suggests that the NPAR index may be one of the influencing factors of biological aging.

Most of the existing studies focus on the predictive value of NPAR in the prognosis of specific diseases [19–24], but there are still important gaps in the current research on the association between NPAR and biological aging: the direct association between NPAR and KDMage/PhenoAge has not been systematically verified in the population of internal RA, and its feasibility as an indicator of biological aging still needs to be explored. If NPAR is confirmed as a factor in aging, it can be incorporated into regular health assessments, providing a novel, low‐cost, and efficient method to identify individuals who may require more closely monitored or preventive measures.

## 2. Materials and Methods

### 2.1. Data Sources

The National Health and Nutrition Examination Survey (NHANES) is a nationwide cross‐sectional surveillance program led by the National Center for Health Statistics (NCHS), part of the Centers for Disease Control and Prevention (CDC). The survey adopts a stratified multistage probability sampling strategy, systematically collects multidimensional health data of ordinary residents and nonhospitalized civilians in the United States, and constructs a comprehensive database through standardized questionnaires, clinical examinations and laboratory tests, to provide a core evidence basis for the scientific assessment of the health and nutritional status of the American population. NHANES implements strict ethical norms throughout the process: the study protocol is approved by the NCHS Ethics Review Committee, and all participants sign a written informed consent form. The design and presentation of this study fully followed the Enhanced Epidemiological Observational Studies (STROBE) guidelines. For details of the project methodology and data acquisition, please refer to the official platform: https://www.cdc.gov/nchs/nhanes/.

### 2.2. Study Population

**Figure 1:**
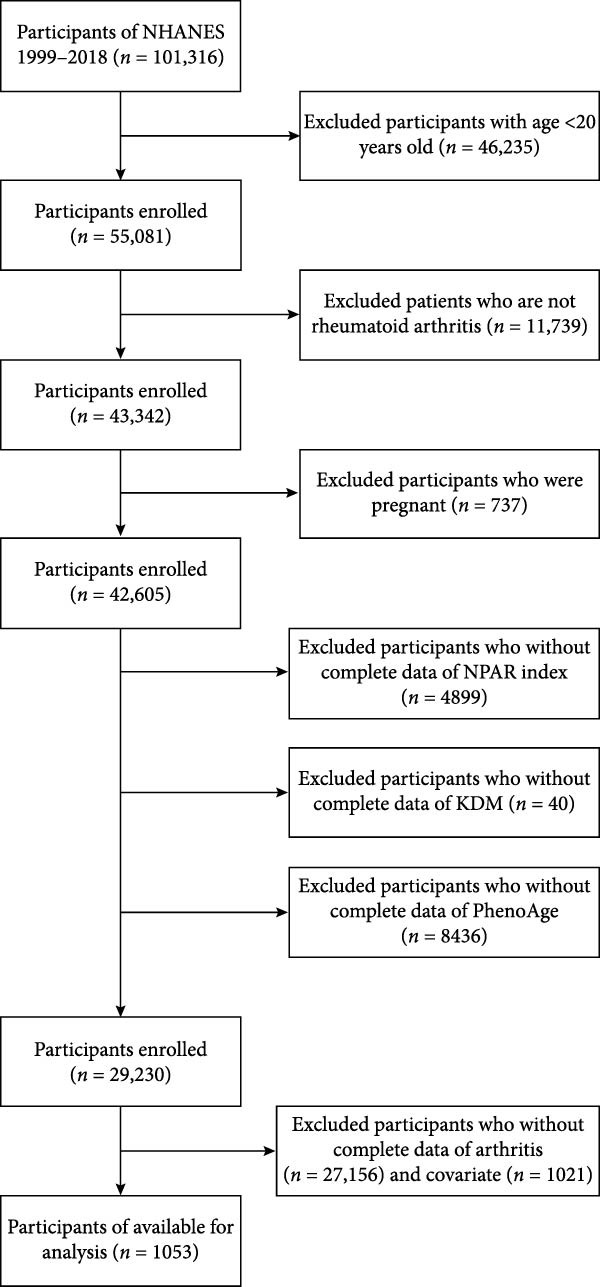
Researcher inclusion and exclusion flow chart.

Our analysis used data from 10 cycles of NHANES (1999–2018) with a total of 1053 eligible participants, with a focus on nonpregnant patients with RA aged 20 years and older. Exclusion criteria included: participants under 20 years of age (*n* = 46235), patients without RA (*n* = 11739), pregnant women (*n* = 737), missing part of NPAR index component data (*n* = 4899), missing elemental data required to calculate BA (*n* = 40) and phenotypic age of the KDM (*n* = 8436), missing arthritis data (*n* = 27156), and smoking status, alcohol consumption status, covariates such as body mass index (BMI), weight information, and demographic data were absent (*n* = 1021). Finally, a total of 1053 participants were included in the study. Figure 1 is a flowchart of the inclusion exclusions selected by the study participants.

### 2.3. Definition of NPAR

In this study, the Beckman Coulter DxH 900 automated blood analysis system (Beckman Coulter, Brea, CA, USA) was used to measure hematological parameters in strict accordance with the NHANES complete blood count (CBC) standard procedure. The device processes the sample through an automated dilution mixing system and integrates impedance cell counting (performing RBC/white blood cell [WBC] count and hematocrit detection) with single‐beam photometer (wavelength 540 nm hemoglobin measurement) technology. The leukocyte differential count is done by Coulter VCS (volume–conductance–light scattering) three‐dimensional analysis technology, and the final output parameters include: neutrophil percentage (%), lymphocyte percentage, and other subset data. Based on the above test results, the neutrophil–albumin ratio (NPAR) was calculated by the formula: NPAR = [Percentage of neutrophils (% of total WBC)]/[albumin concentration (g/dL)] [25].

### 2.4. Definition of Biological Aging

KDM‐Age, or BA calculated based on the KDM, is an algorithm used to assess the BA of an individual. The calculation of BA aims to provide a better indicator of an individual’s aging state than his or her actual age [26, 27]. The KDM is one of the commonly used methods for estimating BA [28]. The calculation does not rely on a single indicator, but combines multiple biomarkers to comprehensively assess an individual’s physiological age through mathematical modeling. The core of this approach is that it believes that aging is the result of a combination of factors, so it is necessary to comprehensively consider the functional indicators of multiple important organs to more accurately assess the overall aging degree [29]. The KDM‐Age calculation is based on the following equation, which takes information from n chronological age regression lines regressed over *n* biomarkers:

KDM-BA=∑i=1nxi−qikisi2+CAsBA2∑i=1nkisi2+1sBA2.

In this study, variable *x* represents the value of a particular biomarker in the individual measurements. For each biomarker included in the analysis, the specific algorithm parameters were expressed as: *i* (regression intercept), *k* (regression slope), *q* (slope parameter), and *s* (root mean square error). The key scale factor sBA is defined as the square root of the actual age (CA) variance interpreted by this collection of biomarkers in the reference sample. The BioAge algorithm package selected nonpregnant participants aged 30–75 years with NHANES III (Third NHANES) as a reference sample, and the parameters of the algorithm were estimated independently by sex (i.e., the parameters were estimated separately for males and females).

Our research adopted a set of 10 biomarkers determined based on previous studies, specifically including: systolic blood pressure (SBP), albumin, alkaline phosphatase (ALP), blood urea nitrogen (BUN), creatinine, glycated hemoglobin (HbA1c), total cholesterol, percentage of lymphocytes, WBC count, and mean corpuscular volume (MCV). It should be noted that we did not use forced expiratory volume in 1 s (FEV1) and C‐reactive protein (CRP), as their measurements were not available during the main data period of this study (2011–12). The BA model used in this study (based on 10 biomarkers) has been validated in previous research. To assess its applicability in the context of this study, we systematically compared its published performance data with the Levine model. The results showed comparable predictive power between the two. Therefore, we selected this model to calculate the BA of the samples [5].

Phenotypic age (PhenoAge) is an indicator used to assess an individual’s biological aging status, equivalent to traditional chronological age, and more accurately reflects an individual’s physiological function and health condition [30–32]. The calculation of phenotypic age aims to quantify the actual rate of aging in individuals and predict age‐related health risks, such as cardiovascular disease and mortality [33]. Phenotypic age is typically calculated using a multivariate regression model based on multiple physiological indicators. This model takes chronological age as the dependent variable and a series of age‐related biomarkers as independent variables. The final equation for calculating phenotypic age is as follows:

Phenotypic age=ln−0.00553×ln−1.51714×expxb0.00769270.09165,

where (*xb*) is calculated as follows: *xb* = − 19.907 − (0.0336 × albumin) + (0.0095 × creatinine) + (0.1953 × glucose) − (0.0120 × lymphocyte percentage) + (0.0268 × mean cell volume) + (0.3306 × erythrocyte distribution width) + (0.00188 × alkaline phosphatase) + (0.0554 × white blood cell count) + (0.0804 + chronological age)

Biological aging is defined as the value of BA greater than chronological age [34], BA acceleration is calculated as the difference between BA estimates and chronological age30, phenotypic age acceleration (PhenoAge_ADVANCE) is calculated as the residuals of chronological age regression PhenoAge, and KDM acceleration (KDM_ADVANCE_) =  KDM biological age– chronological age, all used to measure the rate of aging. A positive age acceleration value indicates that the BA of an individual is higher than their actual age, indicating that it is aging faster. A negative accelerated value of age indicates that an individual’s BA is lower than their chronological age, indicating a relatively slower rate of aging.

This study uses the method of Huang et al. [35], and the method description partly reproduces their wording. KDM BA and phenotypic age are constructed using the R software package BioAge (https://github.com/dayoonkwon/BioAge).

### 2.5. Covariates

Based on previous studies, covariates included in this study included: age, sex, ethnicity, education level, marital status, poverty–income ratio, smoking status, alcohol consumption status, physical activity, BMI, hypertension, diabetes, and cancer, and these data were collected through a baseline questionnaire administered by trained professionals. Participants provided information on age, gender (male or female), and ethnicity (Mexican–American, other Hispanic, non‐Hispanic white, non‐Hispanic black, and other races). Marital status is classified as married, widowed, divorced, separated, unmarried, and cohabitation with a partner. Educational attainment is classified as lower than high school education, high school or equivalent, university, and above. The poverty‐to‐income ratio (PIR) was divided into <1.3, 1.3–3.5, and > 3.5. Smoking status is classified as current smoking, former smoker, or never‐smoker, while drinking status is classified as yes or no. Calculate the BMI (weight [kg]/height [m^2^]) and divide it into <25, 25–30, and ≥30. Physical activity was assessed using task metabolic equivalents (METs). Diagnosis of hypertension is based on a history of hypertension with SBP ≥ 140 mmHg and diastolic blood pressure ≥ 90 mmHg. Diabetes mellitus is defined as a history of diabetes mellitus, HbA1c ≥6.5%, fasting blood glucose ≥ 126 mg/dL or 200 mg/dL 2 h postprandial blood glucose, or use of diabetes medications or insulin.

### 2.6. Statistical Analysis

According to the NPAR index level of the participants, NPAR is used as a continuous variable and a classification variable (divided into T1 (NPAR ≤12.85), T2 (12.85 < NPAR ≤15.09), and T3 (NPAR > 15.09) according to the three‐digit number). To evaluate the relationship between NPAR and each group and biological aging and its acceleration, we use multivariate linear regression, logistic regression, threshold effect analysis, and subgroup analysis. And nonlimiting spline curves were analyzed to determine the relationship between the *β* coefficient or advantage ratio (OR) and the 95% confidence interval (CI; 95%) NPAR index and KDM biological/phenotypic age and their respective age acceleration. Model 1 is a rough model that does not include adjustments for any potential confounding factors. Model 2 adjusts for age and gender. Model 3 was further adjusted for race, marital status, education level, and PIR. Model 4 includes additional adjustments for BMI, smoking status, alcohol consumption, physical activity, high blood pressure, diabetes, and cancer. To explore the potential nonlinear dose–response relationship between NPAR index and biological aging (KDM biological/phenotypic age and its respective acceleration), we adopted a restricted cubic spline (RCS) model and adjusted the variables in Model 4. As a continuous variable, the NPAR index is set in four sections at the 5th, 35th, 65th, and 95th percentiles. Based on the smooth curve, we used a two‐segmented linear regression and a logical regression model to identify any threshold effects and adjust for potential confounding factors. Subgroup analysis was carried out according to age, gender, BMI, smoking, alcohol consumption, hypertension, and diabetes, and model 4 was adjusted. In addition, the subject work characteristic (ROC) curve was used to evaluate the ability of NPAR to predict the risk of biological aging acceleration, and the calculation result was the AUC.

To assess the potential linear association between NPAR and biological senescence and its acceleration, sensitivity analyses were performed: additional analyses were performed after imputing missing information for covariates.

All analyses used R version 4.4.2 (http://www.R-project.org,R, *R*, R Foundation), and *p*‐value of 0.05 or less is considered statistically significant.

## 3. Results

As shown in Table 1, we included 1053 participants, who were evenly divided into three groups (T1: 345 people, T2: 351 people, and T3: 357 people) according to the three‐thirds of NPAR. Women accounted for 56.48%, non‐Hispanic whites accounted for 70.23%, and the proportion of obese (BMI ≥ 30) people was the highest (45.36%). Nearly half (49.37%) have a university degree or above. Comparison between groups showed that the positivity rates of T3 > 65‐year‐old people (34.75%), women (62.80%), diabetes (27.69%), and aging indicators (KDM 42.81% and PhenoAge 62.13%) were significantly higher than those of the other two groups (*p* ≤ 0.047), while BMI, race, and marriage were significantly higher than those of the other two groups (*p* ≤ 0.047). There was no statistical difference in the distribution between variable groups such as education (*p* > 0.05). It is worth noting that the proportion of obesity and hypertension in the whole sample is higher (45.36% of obesity and 56.48% of hypertension), and T3 shows more prominent aging and abnormal metabolism characteristics, suggesting that it is more likely to experience accelerated biological aging.

**Table 1:** Population characteristics by NPAR index.

Variable	Total (*n* = 1053)	T1 (*n* = 345)	T2 (*n* = 351)	T3 (*n* = 357)	Statistic	*p*
Age, *n* (%)					*χ* ^2^ = 11.90	0.017
≤65	663 (71.99)	223 (75.81)	225 (74.90)	215 (65.25)		
> 65	390 (28.01)	122 (24.19)	126 (25.10)	142 (34.75)		
Gender, *n* (%)					*χ* ^2^ = 10.28	0.047
Male	461 (43.52)	162 (49.18)	148 (44.22)	151 (37.20)		
Female	592 (56.48)	183 (50.82)	203 (55.78)	206 (62.80)		
Race, *n* (%)					*χ* ^2^ = 13.69	0.103
Mexican–American	160 (5.43)	46 (4.34)	64 (5.99)	50 (5.93)		
Other Hispanic	101 (5.74)	34 (5.87)	30 (4.49)	37 (6.89)		
Non‐Hispanic White	473 (70.23)	125 (66.02)	164 (71.28)	184 (73.35)		
Non‐Hispanic Black	284 (14.69)	125 (18.86)	82 (14.23)	77 (11.01)		
Other race ‐ including multiracial	35 (3.91)	15 (4.90)	11 (4.02)	9 (2.83)		
Education, *n* (%)					*χ* ^2^ = 5.65	0.528
Less than high school	346 (21.94)	118 (24.35)	116 (22.02)	112 (19.48)		
High school or equivalent	261 (28.69)	73 (25.14)	87 (28.22)	101 (32.68)		
College or above	446 (49.37)	154 (50.51)	148 (49.76)	144 (47.84)		
Marriage, *n* (%)					*χ* ^2^ = 18.66	0.312
Married	559 (59.82)	183 (61.67)	189 (58.39)	187 (59.44)		
Widowed	155 (10.51)	47 (8.75)	48 (9.96)	60 (12.81)		
Divorced	168 (14.67)	51 (11.66)	65 (18.81)	52 (13.46)		
Separated	43 (3.26)	15 (3.22)	16 (3.91)	12 (2.64)		
Never married	83 (7.34)	33 (10.43)	19 (5.07)	31 (6.57)		
Living with partner	45 (4.40)	16 (4.28)	14 (3.86)	15 (5.08)		
Poverty income ratio (PIR), *n* (%)					*χ* ^2^ = 11.20	0.129
<1.3	377 (25.39)	119 (24.26)	126 (25.20)	132 (26.71)		
1.3–3.5	415 (37.78)	130 (32.27)	140 (39.92)	145 (41.07)		
> 3.5	261 (36.83)	96 (43.47)	85 (34.88)	80 (32.22)		
Smoking, *n* (%)					*χ* ^2^ = 2.53	0.867
Never	428 (37.79)	140 (37.32)	142 (40.00)	146 (36.01)		
Former	367 (34.23)	116 (32.57)	131 (33.63)	120 (36.49)		
Now	258 (27.98)	89 (30.11)	78 (26.37)	91 (27.50)		
Drinking alcohol, *n* (%)					*χ* ^2^ = 4.30	0.278
No	375 (32.29)	115 (28.07)	133 (34.87)	127 (33.84)		
Yes	678 (67.71)	230 (71.93)	218 (65.13)	230 (66.16)		
Metabolic equivalent of task (MET), *n* (%)					*χ* ^2^ = 1.26	0.719
Low physical activity	487 (40.83)	157 (38.67)	154 (40.97)	176 (42.84)		
High physical activity	566 (59.17)	188 (61.33)	197 (59.03)	181 (57.16)		
BMI, *n* (%)					*χ* ^2^ = 14.39	0.084
<25	229 (24.21)	83 (25.88)	69 (20.59)	77 (26.24)		
25–30	323 (30.43)	125 (35.28)	109 (31.34)	89 (24.69)		
≥30	501 (45.36)	137 (38.84)	173 (48.07)	191 (49.08)		
Hypertension, *n* (%)					*χ* ^2^ = 2.51	0.563
No	378 (43.52)	124 (42.60)	128 (46.81)	126 (41.11)		
Yes	675 (56.48)	221 (57.40)	223 (53.19)	231 (58.89)		
Diabetes, *n* (%)					*χ* ^2^ = 15.21	0.004
No	738 (77.21)	261 (84.19)	246 (75.20)	231 (72.31)		
Yes	315 (22.79)	84 (15.81)	105 (24.80)	126 (27.69)		
KDM advance, *n* (%)					*χ* ^2^ = 24.35	0.004
No	713 (66.94)	259 (74.19)	248 (69.46)	206 (57.19)		
Yes	340 (33.06)	86 (25.81)	103 (30.54)	151 (42.81)		
PhenoAge ADVANCE, *n* (%)					*χ* ^2^ = 54.50	<0.001
No	518 (53.92)	207 (62.26)	195 (61.61)	116 (37.87)		
Yes	535 (46.08)	138 (37.74)	156 (38.39)	241 (62.13)		

### 3.1. The Relationship Between NPAR and Biological Aging

Tables 2 and 3 show a significant positive correlation between NPAR and biological aging, with each unit increase in NPAR associated with an increased risk of biological aging in all models (*p*  < 0.05). After adjusting for all confounders in Model 4, a 1‐unit increase in the NPAR index was associated with a 0.86‐year increase in KDM BA and a 15% increase in the risk of accelerated aging. Similarly, for every 1 unit increase in the NPAR index, phenotypic age increased by 1.32 years. 24% increased risk of leading to accelerated aging. When classifying the NPAR index, the T3 group had a 149% increased risk of accelerated aging based on BA and a 259% increase in the risk of accelerated aging based on phenotypic age in the T1 group.

**Table 2:** The association between NPAR index and KDM biological age/phenotypic age.

Variables	Model 1		Model 2		Model 3		Model 4	
	*β* (95% CI)	*p*	*β* (95% CI)	*p*	*β* (95% CI)	*p*	*β* (95% CI)	*p*
KDM biological age								
NPAR	1.13 (0.57–1.69)	<0.001	0.86 (0.34–1.39)	0.002	0.93 (0.41–1.46)	<0.001	0.86 (0.39–1.32)	<0.001
NPAR tertiles								
T1	0.00 (reference)	—	0.00 (reference)	—	0.00 (reference)	—	0.00 (reference)	—
T2	3.56 (0.54–6.58)	0.023	3.42 (0.48–6.35)	0.025	3.43 (0.28–6.58)	0.036	2.93 (−0.22 to 6.07)	0.072
T3	8.84 (4.99–12.69)	<0.001	6.89 (3.23–10.55)	<0.001	7.37 (3.80–10.95)	<0.001	6.54 (3.44–9.65)	<0.001
Phenotypic age								
NPAR	1.58 (1.12–2.03)	<0.001	1.36 (0.97–1.74)	<0.001	1.42 (1.02–1.82)	<0.001	1.32 (0.93–1.71)	<0.001
NPAR tertiles								
T1	0.00 (reference)	—	0.00 (reference)	—	0.00 (reference)	—	0.00 (reference)	—
T2	2.84 (0.09–5.58)	0.045	2.90 (0.60–5.20)	0.015	2.84 (0.44–5.24)	0.023	2.06 (−0.24 to 4.35)	0.083
T3	10.39 (7.42–13.36)	<0.001	8.71 (6.34–11.08)	<0.001	9.22 (6.77–11.68)	<0.001	8.29 (5.87–10.70)	<0.001


*Note*: Model 1: crude. Model 2: adjust for the following confounding factors: age and gender. Model 3: adjust for the following confounding factors: age, gender, race, education level, marital status, and poverty–income ratio (PIR). Model 4: adjust for the following confounding factors: age, gender, race, education level, marital status, poverty–income ratio (PIR), smoking status, alcohol consumption, physical activity, BMI, hypertension, diabetes, and cancer history.

Abbreviation: CI, confidence interval.

**Table 3:** The association between NPAR index and KDM biological age/phenotypic age acceleration.

Variables	Model 1		Model 2		Model 3		Model 4	
	OR (95% CI)	*p*	OR (95% CI)	*p*	OR (95% CI)	*p*	OR (95% CI)	*p*
KDM biological age								
NPAR	1.11 (1.03–1.20)	0.005	1.12 (1.04–1.21)	0.004	1.14 (1.04–1.24)	0.004	1.15 (1.05–1.25)	0.003
NPAR tertiles								
T1	1.00 (reference)	—	1.00 (reference)	—	1.00 (reference)	—	1.00 (reference)	—
T2	1.26 (0.76–2.11)	0.374	1.27 (0.76–2.14)	0.360	1.31 (0.76–2.27)	0.332	1.30 (0.72–2.34)	0.382
T3	2.15 (1.37–3.39)	0.001	2.26 (1.42–3.61)	<0.001	2.46 (1.45–4.18)	0.001	2.49 (1.44–4.31)	0.002
Phenotypic age								
NPAR	1.17 (1.09–1.26)	<0.001	1.21 (1.12–1.30)	<0.001	1.24 (1.15–1.34)	<0.001	1.24 (1.15–1.34)	<0.001
NPAR tertiles								
T1	1.00 (reference)	—	1.00 (reference)	—	1.00 (reference)	—	1.00 (reference)	—
T2	1.03 (0.70–1.51)	0.887	1.08 (0.72–1.63)	0.712	1.17 (0.75–1.81)	0.490	1.02 (0.64–1.61)	0.941
T3	2.71 (1.79–4.10)	<0.001	3.22 (2.07–4.98)	<0.001	3.78 (2.33–6.13)	<0.001	3.59 (2.17–5.95)	<0.001


*Note*: Model 1: crude. Model 2: adjust for the following confounding factors: age and gender. Model 3: adjust for the following confounding factors: age, gender, race, education level, marital status, and poverty–income ratio (PIR). Model 4: adjust for the following confounding factors: age, gender, race, education level, marital status, poverty–income ratio (PIR), smoking status, alcohol consumption, physical activity, BMI, hypertension, diabetes, and cancer history.

Abbreviations: CI, confidence interval; OR,: odds ratio.

Non‐restricted spline curve analysis as shown in Figure 2, there is a nonlinear positive correlation between the NPAR index and biological aging, especially the phenotypic age (nonlinear, *p* = 0.05). The reference value of the curve is 13.128. Below 13.128, every increase in the NPAR index by 1 unit is linearly related to biological aging. The growth rate is slower, and above 13.128, the growth rate of biological aging is significantly accelerated, suggesting that the maintenance of NPAR < 13.128 may delay phenotypic aging. The acceleration of phenotypic age (nonlinearity, *p* = 0.002) has a nonlinear relationship, and the reference value of the curve is 14.512. When the NPAR is 14.512, the reference value of the curve is 14.512. At 14.512, the curve fluctuates gently, and the OR value is stable below 1.0 (such as OR = 0.8 at NPAR = 10), indicating that the risk of aging may be reduced when the NPAR is below the threshold; when NPAR > 14.512: the OR value rises sharply (OR = 15.0 at NPAR = 20), 95% CI is out of The 1.0 line indicates that high NPAR levels significantly accelerate phenotypic aging (doubling the risk per unit increase).

Figure 2Nonparametric cubic spline model of the relationship between NPAR index and phenotypic age (a) and phenotypic age acceleration (b). Adjust for the following confounding factors: age, gender, race, education level, marital status, poverty income ratio (PIR), smoking status, alcohol consumption, physical activity, BMI, hypertension, diabetes, and cancer history.

**Figure:**
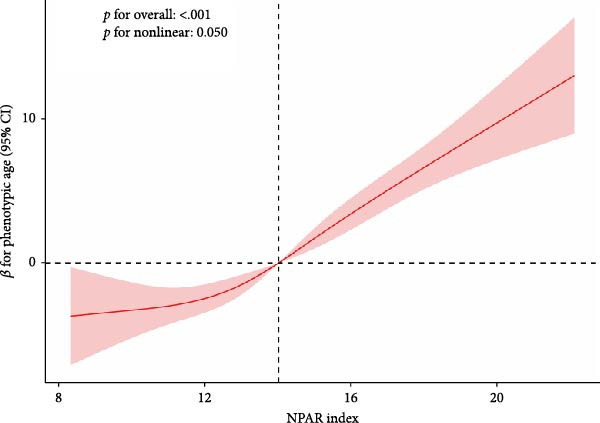
(a)

**Figure:**
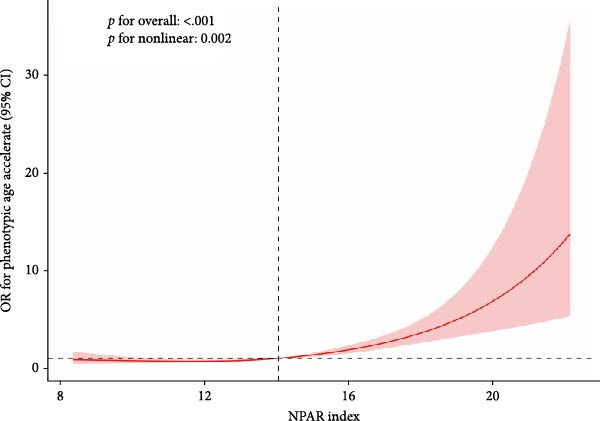
(b)

Perform ROC analysis to evaluate the ability of NPAR to predict the risk of accelerated biological aging. The results, as shown in Figure 3, indicate that the AUC for NPAR’s prediction of the risk of accelerated biological aging (combining demographic, lifestyle, and clinical variables) was 0.71 and 0.75, respectively.

Figure 3Receiver operating characteristic curves predicting KDM biological aging acceleration (a) versus phenotypic age acceleration (b) of NPAR index in adult rheumatoid arthritis patients in the United States. Adjust for the following confounding factors: age, gender, race, education level, marital status, poverty income ratio (PIR), smoking status, alcohol consumption, physical activity, BMI, hypertension, diabetes, and cancer history.

**Figure:**
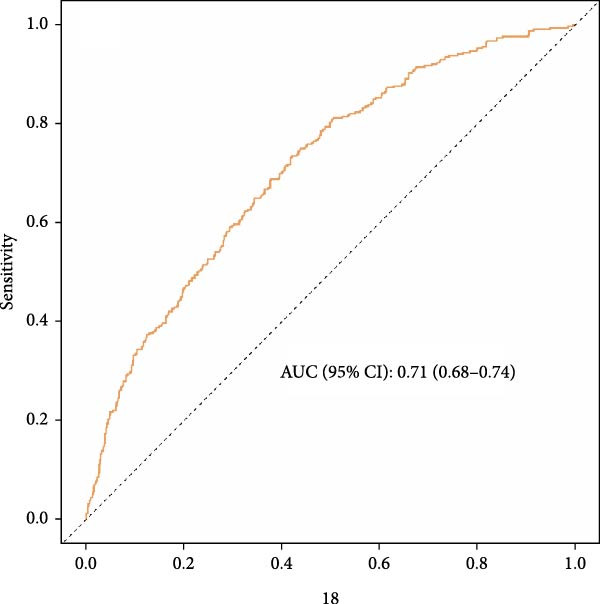
(a)

**Figure:**
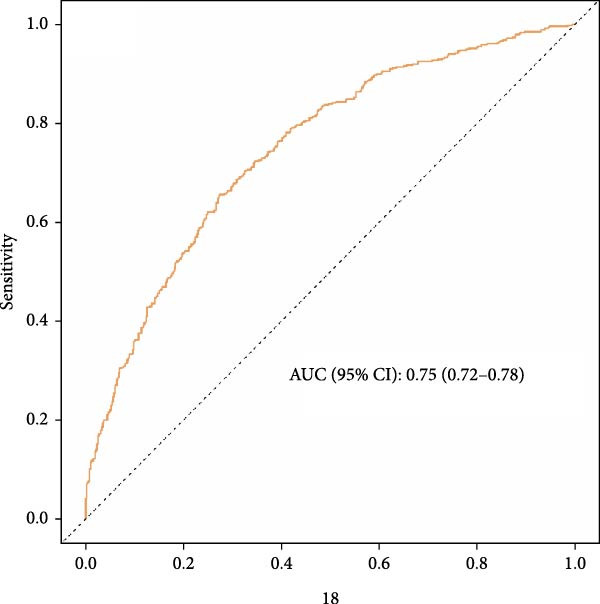
(b)

### 3.2. Subgroup Analysis of NPAR Index and Biological Aging

The subgroup analysis is shown in Figure 4 that there was a significant interaction between gender, smoking status, alcohol consumption, and NPAR (*p*  < 0.05). Specifically, compared with other groups, NPAR of men, former smokers, and drinkers showed a stronger positive correlation with biological aging (*p*  < 0.05). In PhenoAge, NPAR has always shown a significant positive correlation with biological aging in all subgroups.

Figure 4Subgroup analysis of the correlation between the NPAR index and KDM biological age (a) and phenotypic age (b) was adjusted for the following confounding factors: age, gender, race, education level, marital status, poverty income ratio (PIR), smoking status, alcohol consumption, physical activity, BMI, hypertension, diabetes, and cancer history.

**Figure:**
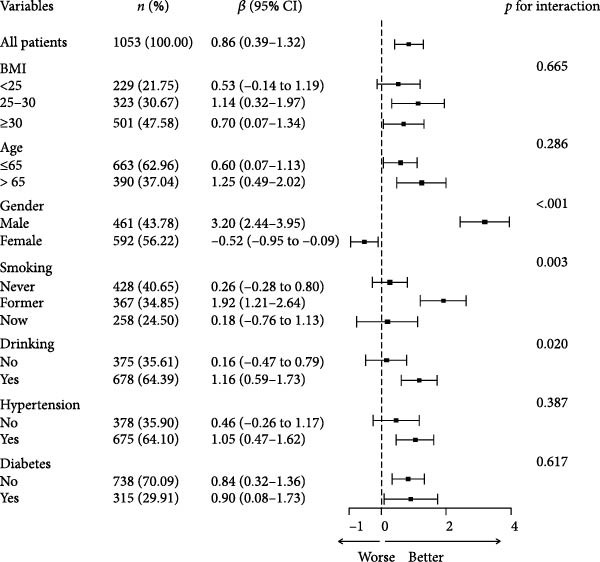
(a)

**Figure:**
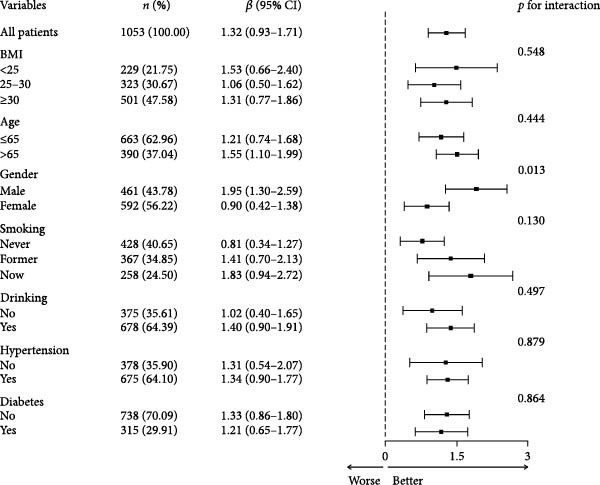
(b)

## 4. Discussion

Based on nationally representative NHANES data, this study demonstrates for the first time an important link between NPAR and biological aging and its acceleration in adult RA patients in the United States. After adjusting for demographic, lifestyle, and clinical factors, higher NPAR values were positively correlated with a higher biological risk of aging. In a cross‐sectional analysis of a national sample, we observed that a higher NPAR index was consistently associated with an increase in biological aging, regardless of the specific definition of biological aging used. The robustness of these findings was confirmed by a sensitivity analysis. These results suggest that NPAR, as an easily available integrated biomarker, may reflect the biological aging process. In addition, smooth curve fitting showed a nonlinear relationship between NPAR index and biological senescence, especially KDM BA (*p* for nonlinear = 0.02) and phenotype age (*p* for nonlinear = 0.05).

Elevated NPAR is typically regarded as a marker of chronic inflammatory status, known as “inflammaging” [36]. In the field of biological aging, several studies have shown that inflammation plays a key role in the aging process [37–39]. Neutrophil percentage is an important indicator of acute inflammatory response, and its persistent elevation reflects a chronic, low‐grade inflammatory state. This long‐standing chronic inflammation can cause damage to tissues and cells, accelerating the biological aging process [40]. Inflammatory cytokines and chemokines promote oxidative stress, leading to DNA damage, protein dysfunction, and cellular aging [40]. For example, senescence‐associated secretory phenotype (SASP) factors secreted by senescent cells induce inflammatory responses, further accelerating tissue damage and the aging process. Albumin synthesis decreases under inflammatory conditions, and it itself has anti‐inflammatory and antioxidant properties. A decline in its levels weakens the body’s protective mechanisms, further exacerbating the pro‐aging effects of inflammation. A study clearly indicates a synergistic association between estimated glucose disposal rate (eGDR, a marker of insulin sensitivity) and NPAR with accelerated biological aging, suggesting that insulin resistance and chronic inflammation together significantly accelerate biological aging, with NPAR reflecting the contribution of chronic inflammation to this process [36].

NPAR is closely associated with the development of various age‐related diseases, which themselves are manifestations or results of accelerated biological aging [41]. Elevated NPAR levels are linked to an increased risk of cardiovascular diseases [42], metabolic disorders [43], and neurological diseases [24, 44, 45]. Increased NPAR indicates a higher risk for these diseases, thereby accelerating an individual’s BA.

Chronic inflammation is also called “inflammatory aging” and is a sign of tissue aging [46]. As an inflammatory marker, NPAR’s elevation may reflect an imbalance in immune system function, leading to overactivation of the inflammatory response. This is consistent with the observed decline in immune function and chronic inflammation during aging, suggesting that inflammation‐nutrient imbalance may be a key mechanism driving biological aging. A study by the team of Huazhong University of Science and Technology based on data from NHANES and UK Biobank showed that the KDMage and PhenoAge acceleration values of RA patients were significantly higher than that of healthy people, and the risk of RA increased by 21% and 46% for every 1‐year‐old acceleration of BA, respectively (*p*  < 0.05). More importantly, there is a significant additive interaction between accelerated aging and RA genetic susceptibility (polygenic risk score), suggesting that biological aging may amplify the influence of genetic background on the onset of RA [7].

The association between NPAR and biological aging may be achieved through the following mechanisms: on the one hand, neutrophil expression of CXCR2 can migrate to the inflammatory site in response to CXCL1, but the expression of CXCR2 in senescent neutrophils is downregulated, resulting in abnormal migration function, not only reduces the anti‐inflammatory effect, but also causes damage to the non‐inflammatory site [47], and low albumin levels weaken the inflammatory site. Antioxidant capacity exacerbates DNA damage and abnormal epigenetic modification; aging‐related stress caused by a series of internal and external stimuli can induce aging through the accumulation of cell damage, which ultimately leads to the occurrence and development of aging‐related diseases [48]. On the other hand, the core indicators in the KDMage and PhenoAge models (such as albumin and CRP) have biological interaction with NPAR, and NPAR may indirectly affect the calculation of BA by regulating the dynamic changes of these markers [16]. For example, as the denominator of NPAR, albumin, the decrease in its level not only directly reduces antioxidant capacity but may also promote the expression of inflammatory aging‐related genes by activating the NF‐kB signaling pathway [49, 50].

This study is the first to explore the important link between the NPAR and biological aging. The NHANES database employs a complex sampling design, ensuring reliable extrapolation to a broader U.S. population and the general population. The research perspective not only has innovative and predictive value but also cost‐effectiveness, providing new evidence for the connection between inflammatory markers and biological aging. However, as a cross‐sectional study, it cannot establish a causal relationship between exposure and outcome and may be influenced by unmeasured variables and a relatively small sample size. The study focuses on clinical markers of biological aging rather than at the molecular or cellular level, although the use of two methods for BA calculation enhances the robustness of the results. Future research should include diverse populations, such as children and those from different regions and health conditions, to further investigate whether this relationship persists.

## 5. Conclusion

In a study targeting American adults with RA, NPAR levels showed a significant correlation with biological aging, specifically manifested as higher NPAR levels being associated with greater BA and higher risk of age acceleration. This suggests that NPAR may serve as a potential clinical marker reflecting the imbalance between inflammation and nutrition, as well as the biological aging process. However, due to the limitations of the cross‐sectional design, this study only indicates an association rather than causation, and its predictive value needs to be validated through prospective studies in the future.

## Ethics Statement

The NHANES are public databases. The patients involved in the database received ethical approval. Users can download relevant data for free for research and publication purposes.

## Disclosure

All authors have read and agreed to the published version of the manuscript.

## Conflicts of Interest

The authors declare no conflicts of interest.

## Author Contributions

**Yangyu Xu:** writing – original draft, conceptualization, formal analysis. **Hong Zhao:** writing – review, conceptualization. **Yuxiang Gao:** data curation, formal analysis. **Li Zhao:** visualization. **Jiannan Han:** writing – review, data curation. **Rong Li:** writing – review, data curation. **Liyun Zhang, Junkang Zhao, and Zewen Wu:** supervision and project administration.

## Funding

This study was supported by the Research and Innovation Team Project for Scientific Breakthroughs at Shanxi Bethune Hospital (Grant 2024AOXIANG02), the Shanxi Province Clinical Research Center for Dermatologic and Immunologic Diseases (Rheumatic diseases), The Science and Technology Innovation Project of Higher Education of Shanxi Province (Grant 2024L100), and The Scientific Research Foundation for Talent Introduction of Shanxi Bethune Hospital (Grant 2023RC27).

## Supporting Information

Additional supporting information can be found online in the Supporting Information section.

## Supporting information


**Supporting Information** Threshold effect analysis of the relationship of NPAR index and KDM biological age. Table S2. Threshold effect analysis of the relationship of NPAR index and phenotypic age. Table S3. Threshold effect analysis of the relationship of NPAR index and KDM biological age acceleration. Table S4. Threshold effect analysis of the relationship of NPAR index and phenotypic age acceleration. Table S5. The association between the NPAR index and KDM biological age/phenotypic age after processing the missing covariate data by imputation. Table S6. The association between the NPAR index and KDM biological age/phenotypic age acceleration after processing the data with missing covariates by imputation. Table S7. The association between the NPAR index and KDM biological age/phenotypic age with different covariates. Table S8. The association between the NPAR index and KDM biological age/phenotypic age acceleration with different covariates. Figure S1. Subgroup analysis of the correlation between the NPAR index and KDM biological age acceleration (a) and phenotypic age acceleration (b) was adjusted for age, sex, ethnicity, education level, marital status, PIR, smoking, alcohol consumption, physical activity, BMI, hypertension, diabetes, and cancer. Figure S2. Nonparametric cubic spline model of the relationship between NPAR index and KDM biological age (a) and KDM biological age acceleration (b), adjusted for age, gender, race, education, marital status, PIR, smoking, alcohol consumption, physical activity, BMI, hypertension, diabetes, and cancer.

## Data Availability

The data that support the findings of this study are available from the corresponding author upon reasonable request.
