# Supplementary material for: Association Between Neutrophil Percentage–Albumin Ratio and Biological Aging in Rheumatoid Arthritis in the United States: A Cross‐Sectional Study of NHANES
Source: Mediators Inflamm. 2026 Jan 16;2026:9987170. doi: 10.1155/mi/9987170 (PMC12811150; doi:10.1155/mi/9987170)
Supplement: Supplementary file 1 — Supporting Information Threshold effect analysis of the relationship of NPAR index and KDM biological age. Table S2. Threshold effect analysis of the relationship of NPAR index and phenotypic age. Table S3. Threshold effect analysis of the relationship of NPAR index and KDM biological age acceleration. Table S4. Threshold effect analysis of the relationship of NPAR index and phenotypic age acceleration. Table S5. The association between the NPAR index and KDM biological age/phenotypic age after processing the missing covariate data by imputation. Table S6. The association between the NPAR index and KDM biological age/phenotypic age acceleration after processing the data with missing covariates by imputation. Table S7. The association between the NPAR index and KDM biological age/phenotypic age with different covariates. Table S8. The association between the NPAR index and KDM biological age/phenotypic age acceleration with different covariates. Figure S1. Subgroup analysis of the correlation between the NPAR index and KDM biological age acceleration (a) and phenotypic age acceleration (b) was adjusted for age, sex, ethnicity, education level, marital status, PIR, smoking, alcohol consumption, physical activity, BMI, hypertension, diabetes, and cancer. Figure S2. Nonparametric cubic spline model of the relationship between NPAR index and KDM biological age (a) and KDM biological age acceleration (b), adjusted for age, gender, race, education, marital status, PIR, smoking, alcohol consumption, physical activity, BMI, hypertension, diabetes, and cancer. [file MI-2026-9987170-s001.docx]

**Table S1.** Threshold effect analysis of the relationship of NPAR index and KDM biological age.

| TyG index | Adjusted Model |  |
| --- | --- | --- |
|  | β (95%CI) | *P*-value |
| <9.892 | -3.15 (-8.07 - 1.77) | 0.220 |
| ≥9.892 | 0.89 (0.51 - 1.27) | <.001 |

Adjust for age, gender, race, education, marital status, PIR, smoke, drinking status, physical activity, BMI, hypertension, diabetes mellitus, cancer.

**Table S2.** Threshold effect analysis of the relationship of NPAR index and phenotypic age.

| TyG index | Adjusted Model |  |
| --- | --- | --- |
|  | β (95%CI) | *P*-value |
| <13.128 | -0.01 (-0.87 - 0.85) | 0.980 |
| ≥13.128 | 1.67 (1.27 - 2.06) | <.001 |

Adjust for age, gender, race, education, marital status, PIR, smoke, drinking status, physical activity, BMI, hypertension, diabetes mellitus, cancer.

**Table S3.** Threshold effect analysis of the relationship of NPAR index and KDM biological age acceleration.

| TyG index | Adjusted Model |  |
| --- | --- | --- |
|  | β (95%CI) | *P*-value |
| <9.892 | 0.00 (0.00 - 18.60) | 0.163 |
| ≥9.892 | 1.18 (1.11 - 1.24) | <.001 |

Adjust for age, gender, race, education, marital status, PIR, smoke, drinking status, physical activity, BMI, hypertension, diabetes mellitus, cancer.

**Table S4.** Threshold effect analysis of the relationship of NPAR index and phenotypic age acceleration.

| TyG index | Adjusted Model |  |
| --- | --- | --- |
|  | β (95%CI) | *P*-value |
| <14.512 | 1.08 (0.96 - 1.20) | 0.197 |
| ≥14.512 | 1.49 (1.29 - 1.72) | <.001 |

Adjust for age, gender, race, education, marital status, PIR, smoke, drinking status, physical activity, BMI, hypertension, diabetes mellitus, cancer.

**Table S5.** The association between the NPAR index and KDM biological age/phenotypic age after processing the missing covariate data by imputation.

| Variables | Model1 | |  | Model2 | |  | Model3 | |  | Model4 | |
| --- | --- | --- | --- | --- | --- | --- | --- | --- | --- | --- | --- |
|  | β (95%CI) | *P* |  | β (95%CI) | *P* |  | β (95%CI) | *P* |  | β (95%CI) | *P* |
| KDM biological age |  |  |  |  |  |  |  |  |  |  |  |
| NPAR | 1.34 (0.85 ~ 1.84) | <.001 |  | 0.99 (0.53 ~ 1.44) | <.001 |  | 1.03 (0.57 ~ 1.49) | <.001 |  | 0.90 (0.45 ~ 1.34) | <.001 |
| NPAR tertiles |  |  |  |  |  |  |  |  |  |  |  |
| T1 | 0.00 (Reference) |  |  | 0.00 (Reference) |  |  | 0.00 (Reference) |  |  | 0.00 (Reference) |  |
| T2 | 4.42 (1.80 ~ 7.04) | 0.001 |  | 3.34 (1.17 ~ 5.52) | 0.003 |  | 3.26 (0.91 ~ 5.61) | 0.008 |  | 2.69 (0.20 ~ 5.19) | 0.037 |
| T3 | 8.19 (4.43 ~ 11.94) | <.001 |  | 5.77 (2.55 ~ 8.99) | <.001 |  | 5.99 (2.80 ~ 9.18) | <.001 |  | 4.91 (1.67 ~ 8.15) | 0.004 |
| Phenotypic age |  |  |  |  |  |  |  |  |  |  |  |
| NPAR | 1.64 (1.29 ~ 1.98) | <.001 |  | 1.29 (0.98 ~ 1.59) | <.001 |  | 1.36 (1.06 ~ 1.66) | <.001 |  | 1.25 (0.96 ~ 1.55) | <.001 |
| NPAR tertiles |  |  |  |  |  |  |  |  |  |  |  |
| T1 | 0.00 (Reference) |  |  | 0.00 (Reference) |  |  | 0.00 (Reference) |  |  | 0.00 (Reference) |  |
| T2 | 4.64 (2.49 ~ 6.79) | <.001 |  | 3.66 (1.88 ~ 5.45) | <.001 |  | 3.73 (1.90 ~ 5.57) | <.001 |  | 3.07 (1.38 ~ 4.76) | <.001 |
| T3 | 10.02 (7.72 ~ 12.31) | <.001 |  | 7.56 (5.66 ~ 9.47) | <.001 |  | 7.98 (6.13 ~ 9.82) | <.001 |  | 7.14 (5.41 ~ 8.87) | <.001 |
| CI: Confidence Interval | | | | | | | | | | | |
| Model1: Crude | | | | | | | | | | | |
| Model2: Adjust for age, gender | | | | | | | | | | | |
| Model3:Adjust for age, gender, race, education, marital status, PIR | | | | | | | | | | | |
| Model4: Adjust for age, gender, race, education, marital status, PIR, smoke, drinking status, physical activity, BMI, hypertension, diabetes mellitus, cancer | | | | | | | | | | | |

**Table S6.** The association between the NPAR index and KDM biological age/phenotypic age acceleration after processing the data with missing covariates by imputation.

| Variables | Model1 | |  | Model2 | |  | Model3 | |  | Model4 | |
| --- | --- | --- | --- | --- | --- | --- | --- | --- | --- | --- | --- |
|  | OR (95%CI) | *P* |  | OR (95%CI) | *P* |  | OR (95%CI) | *P* |  | OR (95%CI) | *P* |
| KDM biological age |  |  |  |  |  |  |  |  |  |  |  |
| NPAR | 1.08 (1.03 ~ 1.13) | 0.001 |  | 1.09 (1.04 ~ 1.14) | <.001 |  | 1.10 (1.05 ~ 1.15) | <.001 |  | 1.09 (1.04 ~ 1.16) | 0.002 |
| NPAR tertiles |  |  |  |  |  |  |  |  |  |  |  |
| T1 | 1.00 (Reference) |  |  | 1.00 (Reference) |  |  | 1.00 (Reference) |  |  | 1.00 (Reference) |  |
| T2 | 1.40 (1.04 ~ 1.88) | 0.030 |  | 1.43 (1.06 ~ 1.94) | 0.021 |  | 1.50 (1.10 ~ 2.05) | 0.011 |  | 1.46 (1.04 ~ 2.04) | 0.029 |
| T3 | 1.62 (1.21 ~ 2.16) | 0.002 |  | 1.71 (1.26 ~ 2.33) | <.001 |  | 1.79 (1.31 ~ 2.43) | <.001 |  | 1.68 (1.19 ~ 2.38) | 0.004 |
| Phenotypic age |  |  |  |  |  |  |  |  |  |  |  |
| NPAR | 1.18 (1.12 ~ 1.25) | <.001 |  | 1.20 (1.13 ~ 1.27) | <.001 |  | 1.23 (1.16 ~ 1.31) | <.001 |  | 1.22 (1.15 ~ 1.30) | <.001 |
| NPAR tertiles |  |  |  |  |  |  |  |  |  |  |  |
| T1 | 1.00 (Reference) |  |  | 1.00 (Reference) |  |  | 1.00 (Reference) |  |  | 1.00 (Reference) |  |
| T2 | 1.36 (0.97 ~ 1.90) | 0.074 |  | 1.43 (1.01 ~ 2.02) | 0.045 |  | 1.57 (1.09 ~ 2.27) | 0.018 |  | 1.41 (0.96 ~ 2.06) | 0.086 |
| T3 | 2.83 (2.00 ~ 4.00) | <.001 |  | 3.07 (2.13 ~ 4.41) | <.001 |  | 3.45 (2.33 ~ 5.10) | <.001 |  | 3.28 (2.18 ~ 4.96) | <.001 |
| OR: Odds Ratio, CI: Confidence Interval | | | | | | | | | | | |
| Model1: Crude | | | | | | | | | | | |
| Model2: Adjust for age, gender | | | | | | | | | | | |
| Model3:Adjust for age, gender, race, education, marital status, PIR | | | | | | | | | | | |
| Model4: Adjust for age, gender, race, education, marital status, PIR, smoke, drinking status, physical activity, BMI, hypertension, diabetes mellitus, cancer | | | | | | | | | | | |

**Table S7.** The association between the NPAR index and KDM biological age/phenotypic age with different covariates.

| Variables | Model1 | |  | Model2 | |  | Model3 | |  | Model4 | |
| --- | --- | --- | --- | --- | --- | --- | --- | --- | --- | --- | --- |
|  | β (95%CI) | *P* |  | β (95%CI) | *P* |  | β (95%CI) | *P* |  | β (95%CI) | *P* |
| KDM biological age |  |  |  |  |  |  |  |  |  |  |  |
| NPAR | 1.45 (0.60 ~ 2.30) | 0.001 |  | 1.25 (0.43 ~ 2.06) | 0.004 |  | 1.35 (0.58 ~ 2.12) | 0.001 |  | 1.16 (0.52 ~ 1.81) | 0.001 |
| NPAR tertiles |  |  |  |  |  |  |  |  |  |  |  |
| T1 | 0.00 (Reference) |  |  | 0.00 (Reference) |  |  | 0.00 (Reference) |  |  | 0.00 (Reference) |  |
| T2 | 4.45 (-0.19 ~ 9.09) | 0.065 |  | 5.26 (0.68 ~ 9.84) | 0.028 |  | 5.53 (1.20 ~ 9.85) | 0.016 |  | 5.69 (1.98 ~ 9.40) | 0.005 |
| T3 | 9.82 (3.73 ~ 15.92) | 0.002 |  | 8.48 (2.68 ~ 14.29) | 0.006 |  | 9.54 (4.33 ~ 14.75) | <.001 |  | 7.50 (2.80 ~ 12.20) | 0.003 |
| Phenotypic age |  |  |  |  |  |  |  |  |  |  |  |
| NPAR | 1.51 (0.93 ~ 2.09) | <.001 |  | 1.32 (0.85 ~ 1.80) | <.001 |  | 1.43 (0.99 ~ 1.87) | <.001 |  | 1.28 (0.88 ~ 1.68) | <.001 |
| NPAR tertiles |  |  |  |  |  |  |  |  |  |  |  |
| T1 | 0.00 (Reference) |  |  | 0.00 (Reference) |  |  | 0.00 (Reference) |  |  | 0.00 (Reference) |  |
| T2 | 1.59 (-2.56 ~ 5.75) | 0.455 |  | 2.90 (-0.64 ~ 6.43) | 0.113 |  | 2.83 (-0.75 ~ 6.41) | 0.127 |  | 2.26 (-1.04 ~ 5.56) | 0.188 |
| T3 | 10.19 (6.38 ~ 13.99) | <.001 |  | 9.12 (5.97 ~ 12.28) | <.001 |  | 10.07 (7.19 ~ 12.95) | <.001 |  | 8.47 (5.66 ~ 11.28) | <.001 |
| CI: Confidence Interval | | | | | | | | | | | |
| Model1: Crude | | | | | | | | | | | |
| Model2: Adjust for age, gender | | | | | | | | | | | |
| Model3:Adjust for age, gender, race, education, marital status, PIR | | | | | | | | | | | |
| Model4: Adjust for age, gender, race, education, marital status, PIR, smoke, drinking status, physical activity, BMI, hypertension, diabetes mellitus, cancer,cardiovascular diseases, metabolic syndrome | | | | | | | | | | | |

**Table S8.** The association between the NPAR index and KDM biological age/phenotypic age acceleration with different covariates.

| Variables | Model1 | |  | Model2 | |  | Model3 | |  | Model4 | |
| --- | --- | --- | --- | --- | --- | --- | --- | --- | --- | --- | --- |
|  | OR (95%CI) | *P* |  | OR (95%CI) | *P* |  | OR (95%CI) | *P* |  | OR (95%CI) | *P* |
| KDM biological age |  |  |  |  |  |  |  |  |  |  |  |
| NPAR | 1.12 (1.04 ~ 1.22) | 0.007 |  | 1.15 (1.05 ~ 1.26) | 0.005 |  | 1.18 (1.07 ~ 1.30) | 0.002 |  | 1.17 (1.06 ~ 1.30) | 0.005 |
| NPAR tertiles |  |  |  |  |  |  |  |  |  |  |  |
| T1 | 1.00 (Reference) |  |  | 1.00 (Reference) |  |  | 1.00 (Reference) |  |  | 1.00 (Reference) |  |
| T2 | 1.57 (0.80 ~ 3.10) | 0.194 |  | 1.53 (0.77 ~ 3.03) | 0.227 |  | 1.76 (0.88 ~ 3.52) | 0.118 |  | 1.72 (0.90 ~ 3.27) | 0.106 |
| T3 | 2.23 (1.19 ~ 4.19) | 0.015 |  | 2.49 (1.27 ~ 4.88) | 0.010 |  | 3.02 (1.52 ~ 6.01) | 0.003 |  | 2.76 (1.32 ~ 5.75) | 0.010 |
| Phenotypic age |  |  |  |  |  |  |  |  |  |  |  |
| NPAR | 1.22 (1.12 ~ 1.33) | <.001 |  | 1.25 (1.14 ~ 1.37) | <.001 |  | 1.32 (1.20 ~ 1.46) | <.001 |  | 1.33 (1.20 ~ 1.47) | <.001 |
| NPAR tertiles |  |  |  |  |  |  |  |  |  |  |  |
| T1 | 1.00 (Reference) |  |  | 1.00 (Reference) |  |  | 1.00 (Reference) |  |  | 1.00 (Reference) |  |
| T2 | 1.47 (0.91 ~ 2.37) | 0.123 |  | 1.52 (0.91 ~ 2.56) | 0.115 |  | 1.73 (0.92 ~ 3.24) | 0.094 |  | 1.58 (0.80 ~ 3.14) | 0.196 |
| T3 | 3.65 (2.15 ~ 6.19) | <.001 |  | 4.40 (2.60 ~ 7.45) | <.001 |  | 6.66 (3.52 ~ 12.57) | <.001 |  | 6.38 (3.35 ~ 12.14) | <.001 |
| OR: Odds Ratio, CI: Confidence Interval | | | | | | | | | | | |
| Model1: Crude | | | | | | | | | | | |
| Model2: Adjust for age, gender | | | | | | | | | | | |
| Model3:Adjust for age, gender, race, education, marital status, PIR | | | | | | | | | | | |
| Model4: Adjust for age, gender, race, education, marital status, PIR, smoke, drinking status, physical activity, BMI, hypertension, diabetes mellitus, cancer,cardiovascular diseases, metabolic syndrome | | | | | | | | | | | |


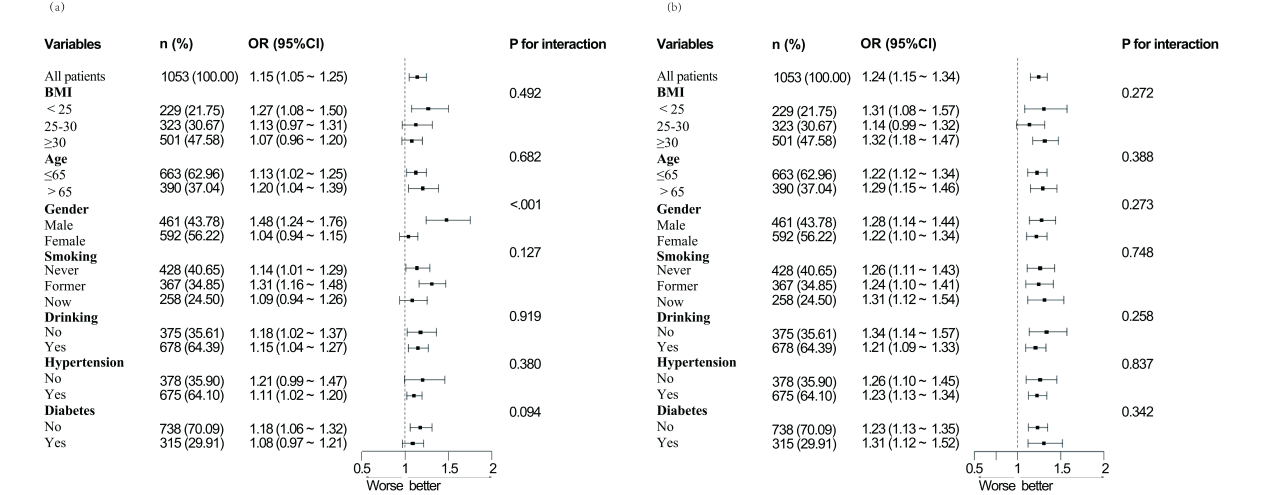


**Figure S1.** Subgroup analysis of the correlation between the NPAR index and KDM biological age acceleration (a) and phenotypic age acceleration (b) was adjusted for age, sex, ethnicity, education level, marital status, PIR, smoking, alcohol consumption, physical activity, BMI, hypertension, diabetes, and cancer.


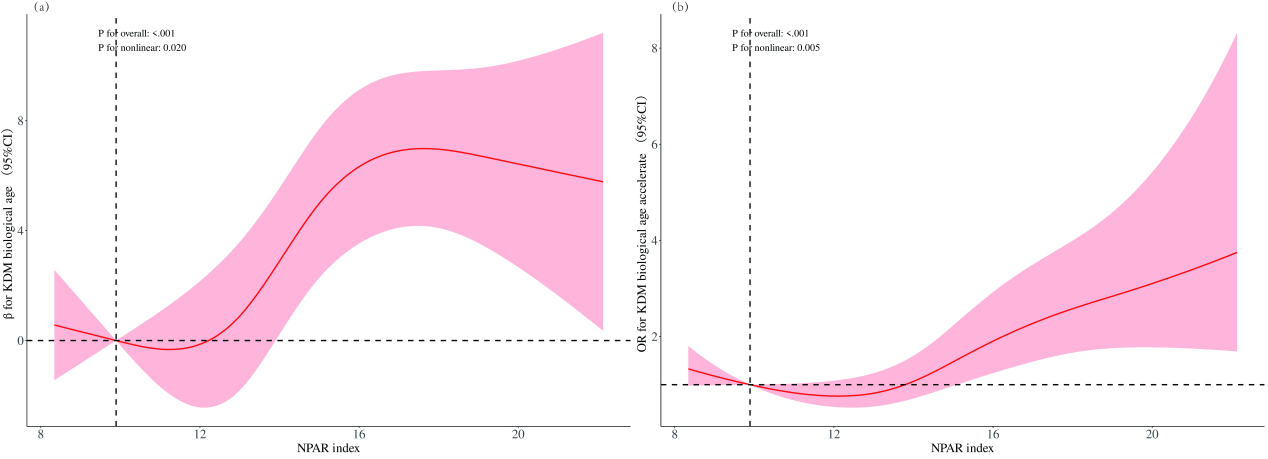


**Figure S2.** Non-parametric cubic spline model of the relationship between NPAR index and KDM biological age (a) and KDM biological age acceleration (b), adjusted for age, gender, race, education, marital status, PIR, smoking, alcohol consumption, physical activity, BMI, hypertension, diabetes, and cancer.
